# Imaging the nanoscale phase separation in vanadium dioxide thin films at terahertz frequencies

**DOI:** 10.1038/s41467-018-05998-5

**Published:** 2018-09-06

**Authors:** H. T. Stinson, A. Sternbach, O. Najera, R. Jing, A. S. Mcleod, T. V. Slusar, A. Mueller, L. Anderegg, H. T. Kim, M. Rozenberg, D. N. Basov

**Affiliations:** 1 Department of Physics, University of California, San Diego, La Jolla, CA 92093 USA; 2 Department of Physics, Columbia University, New York, NY 10027 USA; 3 Laboratoire de Physique des Solides, CNRS, Université Paris-Sud, 91405 Orsay Cedex, France; 4 Electronics and Telecommunications Research Institute, Daejeon, 34129 South Korea; 5 Department of Applied Physics and Materials Science, California Institute of Technology, Pasadena, CA 91125 USA; 6 Department of Physics, Harvard University, Cambridge, MA 02138 USA

## Abstract

Vanadium dioxide (VO_2_) is a material that undergoes an insulator–metal transition upon heating above 340 K. It remains debated as to whether this electronic transition is driven by a corresponding structural transition or by strong electron–electron correlations. Here, we use apertureless scattering near-field optical microscopy to compare nanoscale images of the transition in VO_2_ thin films acquired at both mid-infrared and terahertz frequencies, using a home-built terahertz near-field microscope. We observe a much more gradual transition when THz frequencies are utilized as a probe, in contrast to the assumptions of a classical first-order phase transition. We discuss these results in light of dynamical mean-field theory calculations of the dimer Hubbard model recently applied to VO_2_, which account for a continuous temperature dependence of the optical response of the VO_2_ in the insulating state.

## Abstract (web summary)

The insulator-to-metal transition in vanadium dioxide still has many unexplored properties. Here the authors use multi-modal THz and mid-IR nano-imaging to examine the phase transition in VO_2_ thin films, and discuss the unexpectedly smooth transition at THz frequencies in the context of a dimer Hubbard model.

## Introduction

Vanadium dioxide (VO_2_) is a canonical example of an insulator–metal transition (IMT) material^1,2^ with many exciting potential technological applications^3–5^. Although this material has been studied for decades, the precise physics behind its phase transition is still not fully understood. There is an ongoing debate as to whether the transition is a structurally-driven Peierls-like one, or whether it is Mott-like and due to electronic correlations^6–13^. This complex interplay between Coulomb interaction and structural effects is characteristic of many correlated oxide materials^14,15^.

One major difficulty in disentangling the complexity of VO_2_ physics is that conventional experimental methods for examining the phase transition, such as transport or optical spectroscopy, are necessarily insensitive to the state of the material at short length scales^16,17^. Apertureless scattering near-field optical microscopy (SNOM)^18–23^ has been used to image the electronic phase transition of VO_2_ at mid-infrared frequencies. This technique is able to discriminate between metallic and insulating domains that form near *T*_c_ in VO_2_ thin films^24–26^. These experiments show that VO_2_ phase-separates into coexisting metallic and insulating domains near the transition temperature *T*_c_, consistent with the first-order nature of the phase transition^24–27^.

Here, we use SNOM to investigate the nanoscale optical response through the temperature-driven IMT of VO_2_ films grown on sapphire. Our key innovation is to perform this experiment at both mid-infrared (MIR) and terahertz (THz) frequencies, using a home-built THz-SNOM with a broadband spectrum from 0.1 to 2 THz (see Methods). THz light interrogates the electronic response at energies on the order of meV, which involve excitations very close to the Fermi level and provides a much closer connection to DC transport. The ability to investigate the THz response of VO_2_ with sub-micron spatial resolution allows us to probe the local low-frequency conductivity of individual domains through the transition. We find that the phase transition in the THz appears to be smooth and continuous, in contrast to the abrupt transition at MIR frequencies. We attribute this smoothness in the THz to the gradual filling of the band gap in the insulating state as the system approaches the transition. We find that a dynamical mean-field theory (DMFT) calculation of the dimer Hubbard model (DHM) for VO_2_^28^ qualitatively reproduces our experimental observations.

## Results

### Imaging the IMT in VO_2_ at THz and MIR frequencies

**Fig. 1:**
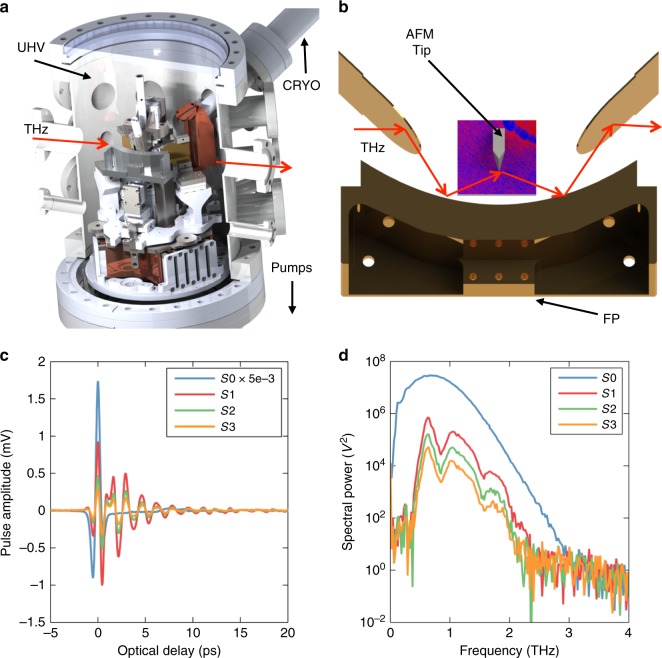
Schematic and typical performance of the THz-SNOM. **a** Schematic of the THz-SNOM. The THz pulse (red arrow) is sent through a UHV vacuum chamber housing the custom SNOM. The sample is coupled to a heater and liquid-He flow cryostat (CRYO), allowing for operation at temperatures from 40 to 400 K. **b** Detail view of the SNOM inside the chamber. The same focusing parabola (FP) is used to both focus the THz pulse onto the tip (not shown to scale) and collect the tip-scattered light. **c** Broadband THz pulse (blue, *S*0) and the near-field THz pulse measured in the system on gold in a dry-air-purged environment. *S*1, *S*2, and *S*3 are the detected THz signal demodulated at the first, second, and third harmonic of the tip tapping frequency. **d** Measured THz spectrum of the far-field pulse (blue) and the near-field spectrum for different harmonics, collected on gold

The principle behind our home-built THz-SNOM is similar in operation to others reported in the literature^29–31^. However, unlike any other THz-SNOM, the sample stage in our system is coupled to a variable temperature liquid helium flow cryostat and situated in ultra-high vacuum (Fig. 1a). This allows for measurements from 30 to 400 K with uncompromised environmental stability which was essential for the measurements we describe below. The spatial resolution of this THz measurement is approximately 130 nm, limited only by the radius of the custom atomic force microscope (AFM) tips used in our apparatus (see Methods).

**Fig. 2:**
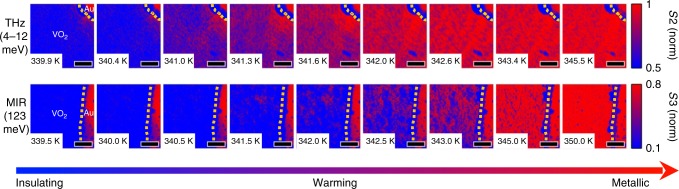
SNOM images of the VO_2_ IMT. The images shown are taken at THz (top row) and MIR (bottom row) frequencies during a heating cycle. The temperature of each image is noted in the bottom left corner. In all images, the signal at every temperature is normalized to the average signal obtained on gold (bright red region in the upper right or right of the image, for THz and MIR respectively). The dashed yellow line denotes the boundary between VO_2_ and gold regions. The THz and MIR data are *S*2 and *S*3, which is the detected signal demodulated at the second and third harmonic of the tip tapping frequency, respectively. Low near-field signal (blue) is measured in insulating regions, while high near-field signal (red) corresponds to a metallic state. The scales are different for the THz and MIR images to highlight the transition from insulator to metal in both cases. Scale bar, 2 μm

In Fig. 2, we show the key experimental data of this work, temperature-dependent images at THz and MIR frequencies of the near-field response of a 100 nm thick VO_2_ film grown on sapphire (see Methods). The top row of images are taken at THz frequencies with our novel instrument as described above, and the images in the bottom row are taken in the MIR with a commercial SNOM (Neaspec GmbH) using a 10 μm CO_2_ laser source. The detected signal *S*_*n*_ is the light scattered by the tip demodulated at the *n*th harmonic of the tip tapping frequency^20^. Contrast in near-field signal *S*_*n*_ has been shown to reliably discriminate between metallic and insulating regions of VO_2_ at MIR frequencies^24–26^, and of other spatially inhomogeneous samples at THz frequencies^29,30^. We detect the amplitude of the peak of the THz pulse scattered by the tip, demodulated at the second harmonic (*S*2) due to limited signal to noise. At THz wavelengths, background contamination of the near-field signal is much less severe, making the second harmonic a good measure of spatially local electromagnetic response^32^. In the MIR images we report *S*3, the tip-scattered light demodulated at the third harmonic of the tapping frequency. Here we choose a linear color scale with red corresponding to high *S*_*n*_ metallic regions, and blue to low *S*_*n*_ insulating regions of the sample. In both the THz and MIR images, the signal *S*_*n*_ is shown normalized to that collected over a region of gold in the same field of view.

**Fig. 3:**
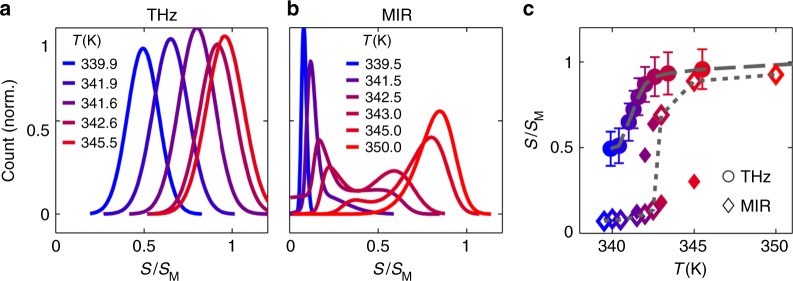
Histogram analysis of the VO_2_ SNOM images at THz and MIR frequencies. **a** Pixel intensity histograms of selected THz images shown in Fig. 2. The signal level *S* is shown normalized to that obtained on gold (*S*_M_). **b** Same, for MIR images. **c** Peak signal level as a function of temperature in the THz (circles) and MIR (diamonds) extracted from single or bi-modal Gaussian fits to the histograms. The error bars (s.d.) in the THz are derived from parameter estimation in a non-linear least-squares fit, and are limited by the random noise in the THz near-field signal measurement. The MIR error bars are smaller than the symbols and so are omitted. In the MIR case, there are two peaks at intermediate temperatures due to the bimodal nature of the pixel intensity distribution. The maximum peak at intermediate temperatures is an open diamond, while the smaller peak is a filled diamond (Supplementary Note 1). The dashed (THz) and dotted (MIR) lines are guides to the eye

The MIR images reveal that upon heating, the VO_2_ sample phase-separates into metallic domains within the insulating background. The metallic regions extend through the sample as temperature is increased. This is similar to previous MIR near-field measurements of VO_2_^24^. In stark contrast, the THz images appear to evolve much more homogeneously and continuously from insulating to metallic signal levels through the same temperature region. A histogram representation of the pixel intensity in each image, as shown in Fig. 3, elucidates this distinction. These histograms exclude the pixels in the gold region of each image. We show histograms of the THz images in Fig. 3a, and histograms of the MIR images in Fig. 3b. At temperatures in the middle of the area-averaged transition, the MIR histograms are bimodal. There is an abrupt change in MIR near-field signal between metallic and insulating domains, represented by the separation between the two peaks in the histograms. The pixels in the THz images, on the other hand, are distributed according to a single Gaussian at all temperatures; there is no clear separation in THz near-field signal between insulating and metallic domains. We can track the average THz near-field signal as a function of temperature by fitting the histograms in Fig. 3a to a single Gaussian distribution and extracting its mean (see Supplementary Note 1 and Supplementary Fig. 1 for details). We plot the mean of each THz histogram as a function of temperature in Fig. 3c as circles, connected by a dashed line as a guide to the eye. The error in the parameter estimation of the fitted histogram is dominated by random noise in the THz-SNOM measurement, which is approximately 5% of the metallic near-field signal level. The THz near-field signal appears to evolve continuously with temperature.

Transport measurements taken with the THz-SNOM apparatus show strong correlation between the transition temperature as revealed by DC transport and *T*_c_ in the THz near-field signal (see Supplementary Note 2 and Supplementary Fig. 2 for details). In addition, the THz near-field signal displays hysteretic behavior, in agreement with a first-order IMT. This evidence indicates that the change in the THz near-field signal between 340 and 350 K is due to a phase transition from an insulating to a fully metallic state. Furthermore, we note that the observed increase in THz near-field signal through the transition is not to be confused with the gradual increase in carrier density before the transition^1^.

There are two important differences between the THz and MIR SNOM measurements. The first is the spatial resolution; the THz-SNOM employs custom AFM probes which have a larger radius than the MIR probes (see Methods). Although the spatial resolution in our THz-SNOM is coarser than that of the MIR-SNOM, autocorrelation analysis of the THz and MIR images shows that the THz-SNOM resolution is sufficient to resolve the inhomogeneity apparent in the MIR images (Supplementary Note 3 and Supplementary Fig. 3). The second and most relevant difference is the variation in near-field signal level between insulating and metallic end states, which is a function of the probing frequency. As we describe below, the THz images are best explained by assuming that the THz near-field signal close to *T*_c_ is very similar in the insulating and metallic state. If the relative signal level between these two phases falls below the THz-SNOM noise floor, then the THz images will not be able to resolve the phase boundary. However, an insulating state whose THz near-field signal is close to that of the metallic state at *T*_c_ implies that the insulating state conductivity is increasing continuously as the temperature approaches *T*_c_.

A closer examination of the MIR near-field images reveals a similar continuous change in signal level. We fit the MIR histograms at temperatures in the middle of the transition to the sum of two skewed Gaussians, whose means are the insulating and metallic signal levels at each temperature (Supplementary Note 1 and Supplementary Fig. 1). For temperatures far from *T*_c_, where the entire image is predominately high or low MIR near-field signal, the MIR histograms could only be fit to a single Gaussian. We plot these extracted mean signals in Fig. 3c as diamonds, of which there are two for temperatures in the middle of the transition. The larger diamond at those temperatures is the center signal of the taller Gaussian, corresponding to the signal level of the majority of pixels (insulating or metallic) in the image. We plot the difference in near-field signal level between the two Gaussian means at intermediate temperatures in Supplementary Fig. 4, which is a good measure of the width of the transition.

We connect the majority signal levels with a dotted line in Fig. 3c, which has an abrupt jump in magnitude at *T*_c_. Although this abrupt jump in the MIR near-field signal between insulating and metallic domains is indicative of a first-order transition, we also observe that the MIR near-field signal within the insulating or metallic phase is changing continuously with temperature below and above *T*_c_. The gradual continuous change in MIR near-field signal of the insulating or metallic state is similar to what we observe in the entire film in the THz, and is contrary to the assumptions of a conventional first-order phase transition. We also note that the signal-to-noise ratio in the MIR-SNOM is significantly higher than that in the THz, such that the error in the parameter estimation is less than the size of the symbols shown in Fig. 3c.

### Nanoscale contrast in the vicinity of the IMT

A first-order phase transition implies an order parameter that changes discontinuously with temperature. In the case of an IMT, that order parameter describes the metallicity of the material, and is closely related to the density of states (DOS) at the Fermi level^33^. In the insulating state the DOS is vanishingly small, and in the metallic state the DOS is finite. As the temperature crosses *T*_c_, the DOS “jumps” from insulating to metallic. Although the MIR near-field images reveal a jump in near-field signal from insulating to metallic, we also observe that the MIR near-field signals in the insulating and metallic state are both temperature dependent. In addition, and rather surprisingly, we observe a homogeneous and continuous evolution of the near-field signal at THz frequencies, which probe the electronic response at energies very close to the Fermi level (Fig. 3a–c). A traditional first-order IMT does not account for these behaviors. Instead, the continuous change in near-field signal evokes a gradual filling of the DOS in the band gap with increasing temperature. This curious discrepancy calls for resolution.

VO_2_ exhibits hysteretic resistance and a divergent molar heat capacity at constant pressure^1,34^, both indicative of a first-order transition. Thus it seems unlikely that the IMT is truly continuous as suggested by the THz near-field images. Another possibility is that a long-range interaction, such as strain, disorder, or even electronic correlations, leads to a micro-emulsion phase whose characteristic domain size is smaller than the spatial resolution of our near-field measurements^35,36^. This latter line of reasoning disagrees with our own observation of clear domain formation of 100–200 nm in the MIR images (Supplementary Note 3 and Supplementary Fig. 3).

Of course, the ability to resolve separate domains depends not only on spatial resolution, but also on the relative signal levels associated with the two states compared to instrumental signal-to-noise. As mentioned above, we interpret the apparent homogeneity of the THz images as most likely due to a reduced THz contrast between the insulating and metallic state at temperatures very close to the transition. The SNOM scattering amplitude is essentially a measure of local reflectivity. Several factors could lead to a high reflectivity in the insulating state, such as a finite or large real part of the dielectric permittivity $\epsilon _1$ of the sample/substrate system. Another possibility for the THz reflectivity in the insulating state being comparable to that of the metallic state is a finite DC conductivity in the insulating state. A small but finite conductivity corresponds to a reflectivity which geometrically increases as the frequency decreases (Supplementary Note 5 and Supplementary Fig. 5). Thus, at THz frequencies films with even a very small optical conductivity in an insulating state would translate into a near-field signal of similar magnitude to that of a metallic state. A finite DC conductivity in the insulating state implies that there is a finite DOS at the Fermi level at temperatures below *T*_c_. Moreover, this qualitative scenario of a gradual filling of the gap can still be consistent with a first-order transition, according to model calculations of a strongly correlated system relevant for VO_2_ that we describe below.

This picture of finite insulating state conductivity near the transition is not necessarily inconsistent with far-field THz studies of VO_2_ films^16,37–39^, which observe a gradual increase in THz conductivity just below *T*_c_. The results of area-averaging far-field experiments and our nano-THz imaging measurements below *T*_c_ where VO_2_ is phase-separated can only be directly compared to each other provided some form of effective medium theory (EMT) is utilized. Any version of the EMT requires multiple inputs including the optical constants of metallic and insulating domains, the filling fractions of the two phases, and also the depolarization factors determined by the shape of the domains^24^. All published attempts of the EMT analysis across the insulator-to-metal transition required assumptions of not only the conductivities but also of the real space characteristics. We note that the nano-scale morphology of VO_2_ in the phase-separated state can widely vary from film to film depending on the substrate material, strain and details of thin film synthesis^40^. Thus, the EMT analysis inevitably hinges on various assumptions that complicate head-to-head comparison of nano-THz data in Fig. 2 and previously reported far-field THz transmission data^16,37–39^. A challenge for future experiments is to perform both far-field THz spectroscopy and nano-THz imaging for the same samples. This is now technically feasible, in principle, and the implementation of this task presents a difficult but attainable goal for future research.

### DMFT solutions of the dimer Hubbard model

An explanation for the formation of in-gap states prior to the metallic transition has an explicit realization in DMFT solutions of the dimer Hubbard model (DHM) (see Methods). The DHM incorporates structural effects into the Mott–Hubbard Hamiltonian by introducing an additional hopping amplitude *t*_⊥_, which accounts for the favorability of intra-dimer interaction relevant to the monoclinic structure of VO_2_^2^. The DHM has been recently recognized to capture non-trivial aspects of the IMT in VO_2_, including a first-order insulator to metal transition with increasing temperature^28^. Moreover, we show below that for certain values of the ratio *t*_⊥_/*U* within the coexistence regime of the model, the DHM predicts both an abrupt, first-order jump in the DOS at *T*_c_ and a gradual, almost continuous filling of the gap at temperatures above and below *T*_c_. These features lead to a qualitative account of our experimental observations.

**Fig. 4:**
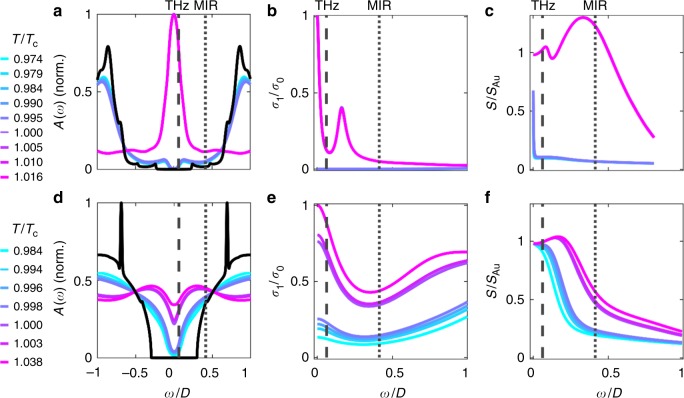
Spectra at different temperatures around *T*_c_, calculated from DMFT for the dimer Hubbard model. Spectra calculated with parameters *t*_⊥_ = 0.2, *U* = 3.1 (**a**–**c**) and *t*_⊥_ = 0.5, *U* = 2.405 (**d**–**f**) as a function of energy normalized to the half-bandwidth *D*. The temperatures (normalized to *T*_c_) of each curve for small *t*_⊥_/*U* (**a**–**c**) are shown to the left of panel (**a**). The temperatures used for large *t*_⊥_/*U* (**d**–**f**) are shown to the left of panel (**d**). The local density of states (LDOS) at different temperatures as a function of energy is shown for small *t*_⊥_/*U* (**a**) and large *t*_⊥_/*U* (**d**). The black line is the LDOS at *T* = 0. The sharp peak in the *T* = 0 LDOS in (**d**) is due to the formation of an intra-dimer singlet at very low temperatures (see text for details). The real part of the optical conductivity at different temperatures is shown for small *t*_⊥_/*U* (**b**) and large *t*_⊥_/*U* (**e**). All conductivities are normalized to the DC conductivity at the highest temperature shown (i.e., the DC conductivity of the metallic state). The calculated near-field signal at different temperatures as a function of frequency is shown for small *t*_⊥_/*U* (**c**) and large *t*_⊥_/*U* (**f**). The vertical gray lines in all figures indicate the THz (dashed) and MIR (dotted) frequencies used for calculating histograms in Fig. 5

In Fig. 4, we show the frequency- and temperature-dependent DOS, optical conductivity, and simulated SNOM signal for two different values of the ratio *t*_⊥_/*U*. Both *t*_⊥_ and *U* are given in units of the half-bandwidth *D* = 2*t*, where *t* is the interdimer lattice hopping amplitude. For VO_2_, *D* ≈ 1 eV. The DOS is calculated from DMFT solutions of the DHM, which in turn is used to calculate the optical conductivity. Finally, we use the resulting optical constants to simulate the near-field signal using a lightning-rod (LR) model of the tip–sample interaction (see Methods and ref. ^41^ for details). Panels (a)–(c) correspond to a case with small *t*_⊥_/*U*, and panels (d)–(f) are for large *t*_⊥_/*U*. The values of *t*_⊥_ and *U* we show are carefully chosen to represent the most physically relevant cases of the model. Both cases are for values of *t*_⊥_/*U* within the coexistence regime of the DHM phase diagram (see Supplementary Fig. 6), meaning that both parameter sets support a first-order temperature-driven IMT with *T*_c_ ≈ 0.04*D* consistent with experiment^28^. The low *t*_⊥_/*U* case represents a model where intra-dimer hopping is relatively weak, similar to a conventional Mott–Hubbard model. The high *t*_⊥_/*U* case corresponds to relatively strong intra-dimer hopping, still within the coexistence region of the phase diagram but closer to the crossover to a Peierls-like transition (see Supplementary Fig. 6). Even though both cases discussed here correspond to a first-order IMT material, the temperature dependence of all three plotted quantities is strongly affected by the value of *t*_⊥_/*U*.

First we consider the small *t*_⊥_/*U* case, which corresponds to a material with strong electronic correlations compared to the intra-dimer hopping amplitude. In Fig. 4a, the DOS exhibits an abrupt shift from insulating to metallic at a specific temperature *T*_c_, but does not display strong temperature dependence at temperatures below or above *T*_c_. This behavior is echoed in the optical conductivity (Fig. 4b); the insulating state has a spectrally flat, insulating conductivity for all temperatures below *T*_c_, which abruptly jumps to a large Drude-like metallic conductivity at all temperatures above *T*_c_. In Fig. 4c, we show the simulated near-field signal on VO_2_ as a function of frequency for different temperatures, which repeats the same general trends as the DOS and the optical conductivity. It is low for *T* < *T*_c_ and high for *T* > *T*_c_, with an abrupt jump in signal level between the two states at all frequencies. There is very little temperature dependence otherwise. We note that the spectra calculated from the DHM are not meant to be quantitative predictions of experiment, but are rather a quasi-qualitative picture of how the near-field spectrum would appear in VO_2_ as described by this simplified model.

The temperature dependence is markedly different for the case of large *t*_⊥_/*U*, which corresponds to a material with strong dimerization compared to the electronic correlations. In Fig. 4d, there is still an abrupt jump in the DOS at *T*_c_, but the DOS below and above *T*_c_ is more temperature dependent. We see that the gap is continuously filling at temperatures below *T*_c_. This is due to the melting of the intra-dimer singlet at low temperature, whose spectral weight is then spread incoherently over the gap as the temperature increases^42^. The transfer of spectral weight over energy scales that are much higher than *T*_c_ is a hallmark of strong correlations^15^. In the present context, it is related to the competition between the intra-dimer screening of the magnetic moments in the insulator and the lattice Kondo-like screening of each lattice site in the metallic state. Namely, the two magnetic sites that screened each other and formed the singlet suddenly experience a qualitative change upon heating and become screened by their respective baths (i.e., Kondo screening). In other words, this feature can be interpreted as a local RKKY-versus-Kondo screening at the level of a single dimer^42–44^. Similarly, in Fig. 4e the optical conductivity at *T* < *T*_c_ is still flat and insulating, but is continuously increasing as temperature increases. There is an abrupt jump in conductivity at *T*_c_ from flat and insulating to Drude-like and metallic, but as temperature continues to rise for *T* > *T*_c_ the conductivity continuously increases. The metallic state of the model at higher *T* corresponds to a (bad) metal controlled by Kondo-like physics, with two parallel quasiparticle bands that are split by an effective *t*_⊥_^28,42^. For the choice of *t*_⊥_/*U* shown in the bottom row of Fig. 4, the low-frequency conductivity changes by an order of magnitude across the transition. This is smaller than the 3–5 orders of magnitude change in DC resistance observed in transport experiments, and is likely due to the fact that our model does not include a structural transition, but nevertheless is consistent with a first-order IMT.

Notably, the insulating state optical conductivity in the high *t*_⊥_/*U* case has a very different behavior at all frequencies with respect to the low-*t*_⊥_/*U* case. The small but finite low-frequency optical conductivity in the insulating state translates to a 1/*ω* behavior in reflectivity, as is expected for a Drude metal whose scattering rate is comparable to the plasma frequency (Supplementary Note 3 and Supplementary Fig. 3). Concurrently, the calculated near-field signal shown in Fig. 4f has a 1/*ω*-like behavior at low temperatures, with a frequency width that is a function of the DC conductivity *σ*_0_ and increases with temperature. Thus, the low-frequency near-field signal has a more continuous and smaller relative change with increasing temperature as the conductivity evolves from insulating to metallic. This is reflected in the modeled near-field signal in Fig. 4f, which shows very little temperature variation at low frequencies, but an abrupt jump with temperature at higher frequencies. As remarked on above, the difference between our THz near-field images on granular thin films and microwave nanoscale images on strained single crystals^40^ might be accounted for by our model as a difference in the value of *t*_⊥_/*U* for disparate sample morphologies.

We wish to emphasize that the DHM model is not a complete description of the phase transition in VO_2_, in that the DHM does not consider the structural transition from monoclinic to rutile. In fact, the metallic state predicted by the DHM is more closely related to that of the so-called “monoclinic metal” reported in previous works^24,26,45–50^. The experimental and theoretical results presented here allow us to form a qualitative description of how the near-field spectrum would appear in a material undergoing an insulator–metal transition with either strong or weak inter-dimer coupling. As such, the DHM is, to first order, a viable model of the VO_2_ system.

### Modeling the VO_2_ near-field imaging experiment

**Fig. 5:**
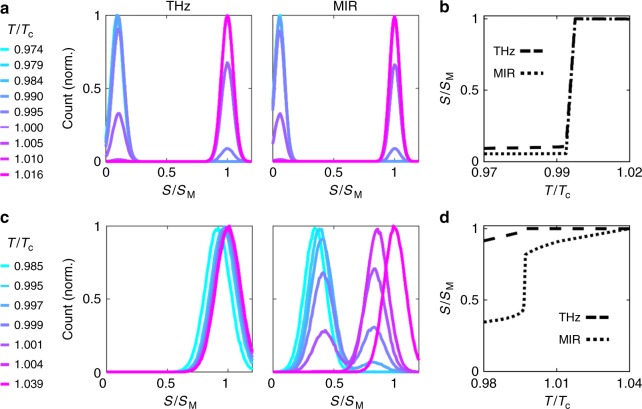
Histograms of a phase-separated image using near-field signals at different temperatures calculated from the DHM. **a** The simulated histograms for a THz and MIR image in the case of small *t*_⊥_/*U*. The signals are calculated at the frequencies represented by the dashed (THz) and dotted (MIR) vertical lines in Fig. 4c. The temperatures shown are normalized to *T*_c_. **b** Plot of the calculated near-field signal as a function of temperature for small *t*_⊥_/*U* at THz (dashed) and MIR (dotted) frequencies. **c** Same as (**a**), but for large *t*_⊥_/*U*. The signals are calculated at the frequencies represented by the dashed (THz) and dotted (MIR) vertical lines in Fig. 4f. **d** Same as (**b**), but for large *t*_⊥_/*U*

To compare the results of the DHM to our experimental data, we use the calculated frequency-dependent near-field signals in Fig. 4c, f to simulate the histogram one would obtain in a near-field imaging experiment (Supplementary Note 6). The simulated histograms for the small *t*_⊥_/*U* case are shown in Fig. 5a. In this case, the temperature dependence of both the THz and MIR near-field signals is similar. The histograms at both frequencies are clearly bimodal. There is a distinct near-field signal level for the insulating and metallic states, with an abrupt jump in signal between the two states. As temperature increases, the near-field signal level in an individual domain does not change, but rather the relative distribution of pixels shifts from majority insulating to majority metallic. The evolution of *S*(*T*) for both THz and MIR frequencies is plotted in Fig. 5b for the small *t*_⊥_/*U* case. At both frequencies, there is an abrupt jump in near-field signal at *T*_c_, but no temperature dependence above or below *T*_c_. Thus, the small *t*_⊥_/*U* case is quite different from what we observe experimentally.

The histograms calculated for large *t*_⊥_/*U*, which are shown in Fig. 5c, qualitatively agree with our experimental data. The THz histograms are single Gaussians with a mean near-field signal level that increases continuously with temperature. The MIR histograms are bimodal with an abrupt jump between the two Gaussian centers, yet still display an insulating and metallic near-field signal level that changes continuously with temperature. This is reflected in the temperature-dependent near-field signal at both frequencies, plotted in Fig. 5d. The THz signal at low temperatures is already very close to the signal at high temperatures, and evolves almost continuously from low to high signal as temperature is increased. The MIR near-field signal, in contrast, has an abrupt jump at *T*_c_, and is also clearly changing with temperature above and below *T*_c_. This is similar to what we observe in the experimentally extracted *S*(*T*) curves shown in Fig. 3c.

## Discussion

Using a novel THz-SNOM with 130 nm spatial resolution, we find that the nature of the domain formation through the phase transition in VO_2_ thin films appears homogeneous and continuous at THz frequencies. Moreover, MIR near-field images reveal that the local reflectivity of the insulating or metallic state is changing with temperature below and above *T*_c_. A relevant additional experiment would be to enhance these data with THz and MIR nano-spectroscopy of the VO_2_ films through the transition. Currently, our apparatus is unable to perform a complete series of nano-spectroscopy measurements across the transition (analogous to the data shown in Fig. 1d) due to tip degradation coupled with the necessary long averaging times. Future developments in instrumentation will hopefully soon overcome these experimental difficulties.

The DHM appears to provide a framework for understanding how a continuously varying electronic response as revealed by the THz and MIR near-field images can be consistent with a first-order transition. Increased intra-dimer hopping with respect to the Coulomb interaction (large *t*_⊥_/*U*) leads to the formation of intra-dimer singlets below a characteristic temperature T^*^^42^. The dissolution of these singlets as *T* approaches *T*_c_ results in an incoherent spread of spectral weight across the gap at finite *T* < *T*_c_. Gap filling in the insulating state at finite temperature is consistent with previous measurements of both bulk and thin film VO_2_^51,52^. This more continuous filling of the DOS for *T* < *T*_c_ leads to a small but finite THz conductivity in the insulating state. A small optical conductivity in turn generates a reflectivity which is low but abruptly increases as the frequency approaches 0. Thus, the increased *t*_⊥_/*U* generates a small, finite THz optical conductivity for *T* ≤ *T*_c_, which translates into an insulating state whose THz reflectivity is larger than the MIR reflectivity. At temperatures very close to the transition, the insulating THz near-field signal is already within the experimental detection limit of the metallic signal. Even though there is an abrupt first-order jump in the optical conductivity at *T*_c_, the relative change in THz near-field signal across the IMT remains below the experimental detection limit. Therefore, the transition appears homogeneous in the THz images. The transition at *T*_c_ from insulating to metallic remains abrupt in the MIR near-field signal, but the continuous filling of the gap translates into a continuous shift of the MIR near-field signal in the insulating and metallic state.

The DHM shows that the first order transition involves a qualitative change of the state, from an insulator with dynamical local singlets to a (bad) metal with the dimer moments independently (Kondo) screened by the rest of the lattice. In this sense, the IMT in VO_2_ is due to Mott physics. However, with regard to previous works utilizing the DHM^42^, the present work seems to indicate that the behavior of VO_2_ may be better characterized by a larger value of *t*_⊥_. In previous works^42^, *t*_⊥_ was 0.3 *W*/*D*, and here we find that the value 0.5 *W*/*D* represents the experiments best. Both values, nevertheless, result in a first-order thermally driven IMT and yield the same underlying physics. The key aspect of the DHM is to explicitly incorporate dimerization into the Hamiltonian, thus merging structural effects of the monoclinic state with a correlation-driven IMT. The success of the DHM in explaining our experiments support the conclusion made by others^10,52,53^ that VO_2_ is neither purely a Mott nor a Peierls insulator, but a hybrid of the two. This interpretation could have profound effects on the understanding of the IMT in related materials such as V_2_O_3_, which also display gradual change of the insulating or metallic state response through the transition^54^.

## Methods

### THz near-field imaging

We use commercially available LT-GaAs photoconductive antennas (PCAs, Neaspec GmbH)^55,56^ as the THz emitter and detector to form a time-domain spectrometer (TDS)^57–59^ based off of a commercially available system (TeraView Ltd.). The PCA emits a broadband pulse with frequencies from 0.1 to 3 THz. We couple that pulse onto a metallic AFM tip (Rocky Mountain Nanotechnology, LLC) whose length is engineered so as to form an antenna resonance with the peak wavelength of the incoming THz pulse. The resonant tip enhances the field at the apex of the tip and increases tip–sample interaction^60^. Our AFM is home-built and incorporates a commercial piezoelectric scanning stage (Attocube Systems). THz light is scattered by the tip into the far field, where it is collected and focused onto the detector PCA. Only frequencies up to 2 THz are efficiently scattered by the tip, limiting the bandwidth of the near-field signal to slightly less than that of the PCA. Detection is identical to conventional THz-TDS, except that the detected THz signal is demodulated at higher order harmonics of the tapping frequency of the tip in order to isolate the near-field component of the scattered light^61^. We measure the scattered amplitude of the peak of the THz pulse, corresponding to a frequency-integrated response over the full bandwidth. We show a typical tip-scattered THz pulse in Fig. 1c and the corresponding spectra in Fig. 1d collected over gold and demodulated at the first, second, and third harmonic of the tip tapping frequency. For comparison we also show the far-field (*S*0) THz pulse and spectrum.

### VO_2_ film growth

The VO_2_ thin films were fabricated on r-cut Al_2_O_3_ substrates by a pulsed laser-deposition method with a 248 nm KrF excimer laser. Prior to the deposition, the chamber was evacuated to a background pressure of ~10^−6^ Torr and the substrate was heated up to 600 °C. To grow the VO_2_ film, a metallic vanadium target was ablated in an oxygen atmosphere at a partial pressure of 30 mTorr. A 30 min deposition process, at the laser pulse energy of 300 mJ and a repetition rate of 10 Hz, yields ~100 nm thick VO_2_ films. Au pads were fabricated on the top of the films, using standard photolithography processes and e-beam evaporation.

### DMFT calculations of the dimer Hubbard model

The theoretical calculations were done on a DHM solved within cluster-dynamical mean-field theory (C-DMFT). This model consists of dimers on each unit cell with intra-dimer hopping, in addition to the standard inter-dimer hopping and a Hubbard-type local Coulomb repulsion. The model Hamiltonian is given by

1 $$H = \left[ { - t\mathop {\sum}\limits_{\langle i,j\rangle \alpha \sigma } {\kern 1pt} c_{i\alpha \sigma }^\dagger c_{j\alpha \sigma } + t_ \bot \mathop {\sum}\limits_{i\sigma } {\kern 1pt} c_{i1\sigma }^\dagger c_{i2\sigma } + {\mathrm{H}}{\mathrm{.c}}{\mathrm{.}}} \right] + \mathop {\sum}\limits_{i\alpha } {\kern 1pt} Un_{i\alpha \uparrow }^\dagger n_{i\alpha \downarrow }$$

where $\left\langle {i,j} \right\rangle$ denotes nearest-neighbor sites, *α* = {1, 2} denote the dimer orbitals, *σ* is the spin, *t* is the inter-dimer (lattice) hopping amplitude, *t*_⊥_ is the intra-dimer hopping amplitude, and *U* is the Coulomb repulsion. The DHM was recently shown to capture a thermally driven insulator-to-metal transition for parameters relevant to VO_2_^28^. It also provides a consistent description of the near-field optical conductivity data across the IMT in that compound.

The model is defined on a semi-circular non-interacting DOS, which is realized on a Bethe lattice^62^. The bandwidth of the model is *W* = 2*D* = 4*t*. The qualitative behavior of the model is not strongly affected by the specific type of lattice adopted. The C-DMFT equations are solved within the iterated perturbation theory (IPT) approximation. At half filling, this approximation is excellent^28,42^. It was found to be asymptotically exact in many cases, including the weak interacting limit (*U* → 0) and in the atomic limit (*t* → 0) for all values of the inter-dimer hopping *t*_⊥_.

We have extensively benchmarked the approximation against exact but more numerically costly quantum Monte Carlo (QMC) calculations. The IPT approximation is found to capture all qualitative features seen in QMC such as a first-order transition and coexistence of solutions for smaller *t*_⊥_ and a continuous transition at higher *t*_⊥_. They also show a similar crossover at higher *T*, and comparison of Green’s functions on the Matsubara axis show very similar behavior. The differences between IPT and QMC solutions of the DHM are similar to the differences of the two methods in the well-studied one band model. Namely, the IPT and QMC solutions differ by about a factor of 2 in the value of the upper finite temperature tip of the coexistence region^28^, and the coexistence region appears somewhat wider within the IPT approximation. Nevertheless, these differences are merely quantitative. Therefore IPT is a very useful approximation for the present study, which remains qualitative or semi-quantitative at most.

Key for our present study, we have implemented a novel finite temperature and real frequency impurity solver, which avoids the technical difficulties of analytic continuation. This allows us to obtain the detailed evolution of the entire DOS with temperature with unprecedented precision.

### Lightning-rod model calculations of near-field signals

For modeling of our near-field data we employed the LR model of probe-sample near-field interaction^41^. The Fresnel reflection coefficient *r*_p_ of the sample for light polarized parallel to the tip axis determines the near-field signal and is evaluated for a VO_2_ single crystal. By formulating the quasi-electrostatic near-field interaction as a scattering problem in momentum space, the LR model generally provides excellent quantitative agreement with near-field spectroscopy measurements at very low computational cost^41^. Experimental details, such as scattering of light from the probe to the detector and demodulation of the detected signal, are included explicitly in the model. Input parameters include the tip radius *a* and tapping amplitude *A*, as well as the overall probe geometry, modeled here as a metallic cone 19 μm in height with half-angle ≈30°, in qualitative accordance with the geometry of commercial probes used for this study. Best results were obtained with *a* = 100 nm and *A* = 200 nm, which agree well with nominal experimental values of *a* ≈ 140 nm and *A* ≈ 250 nm.

### Code availability

The packages used for calculating DMFT solutions are available from M.R. upon reasonable request. The packages used for calculating the lightning-rod model of the near-field signal are available from the corresponding author upon reasonable request.

## Electronic supplementary material

Supplementary Information

## Data Availability

The data supporting the findings of this work are available from the corresponding author upon reasonable request.
