# Supplementary material for: Imaging the nanoscale phase separation in vanadium dioxide thin films at terahertz frequencies
Source: Nat Commun. 2018 Sep 6;9:3604. doi: 10.1038/s41467-018-05998-5 (PMC6127259; doi:10.1038/s41467-018-05998-5)
Supplement: Supplementary file 1 — Supplementary Information [file 41467_2018_5998_MOESM1_ESM.pdf]

Supplementary Information for Stinson, et al., ”Imaging the phase separation in vanadium dioxide thin films at terahertz frequencies”

Suplementary Note 1: Gaussian fits to near-field image histograms

First we obtain histograms of the near-field signal distribution in each THz and MIR image. We crop each image to a 210x125 pixel region that does not contain any of the reference gold. We then split the pixel values into 100 separate bins ranging from the minimum to the maximum pixel signal in each image. Raw histograms are plotted as open symbols in Supplementary Figure 1 for three representative THz (Supplementary Figure 1a) and MIR (Supplementary Figure 1b) images. As mentioned in the main text, the THz histograms are Gaussian at every temperature, while the MIR histograms are bimodal for intermediate temperatures.

To extract an average signal level at each temperature we fit the THz histograms to the skewed Gaussian function

$$Ae^{-\frac{(x-\mu)^2}{2\sigma^2}}\left(1-\mathrm{erf}\left[\frac{\alpha\,(x-\mu)}{\sqrt{2}\sigma}\right]\right) \tag{1}$$

where the four free parameters are the amplitude A, the standard deviation σ, the skew parameter α, and the mean μ. The fitted functions are plotted as solid lines for three representative temperatures in Supplementary Figure 1a. This last parameter μ is what is plotted as a function of temperature in Fig. 3c (open circles) of the main text.

Because the MIR images display phase separation, the histograms near the transition temperature are bimodal. Therefore we fit the MIR histograms to a function for the sum of two skewed

Gaussians:

$$A_1 e^{-\frac{(x-\mu_1)^2}{2\sigma_1^2}} \left(1 - \text{erf}\left[\frac{\alpha_1 (x - \mu_1)}{\sqrt{2}\sigma_1}\right]\right) + A_2 e^{-\frac{(x-\mu_2)^2}{2\sigma_2^2}} \left(1 - \text{erf}\left[\frac{\alpha_2 (x - \mu_2)}{\sqrt{2}\sigma_2}\right]\right) \quad (2)$$

The fitted functions are plotted as solid lines in Supplementary Figure 1b for three representative temperatures. At 339.5 K and 350.0 K, far from the transition temperature $T_c \approx 342$ K, the MIR histograms can be fit with a single skewed Gaussian (Eq. 1). The MIR histograms near T_c are fit with a bimodal function (Eq. 2). Similarly to the THz data, we plot μ_1 and μ_2 in Fig. 3c (diamonds) of the main text. The open diamonds have the same subscript as the maximum of A_1 or A_2; that is, the open diamonds are the average signal value for the majority of pixels in the image. The filled diamonds are the average signal value of the remaining Gaussian component. For temepratures where a single Gaussian fits the histogram, we plot a single filled diamond.

Supplementary Note 2: THz near-field hysteresis and DC transport

In Supplementary Figure 2, we plot the average THz near-field signal over a wide range of temperatures taken in both the heating (red) and cooling (blue) directions of the temperature cycle. The average THz near-field signal at each temperature is extracted from histogram fitting as described in Supplementary Note 1. Although the data for decreasing temperatures does not fully close the hysteresis loop, the hysteretic behavior in the THz near-field signal upon cooling is evident. We were unable to obtain a full set of THz data for decreasing temperatures due to degradation of the AFM tip

used in the experiment. Because the formation of real-space patterns in the phase transition is spontaneous, a full imaging series must be performed in a single shot to compare images at different temperatures. Unfortunately, our setup prohibits the exchange of AFM tips without altering the sample temperature and thus disrupting a cooling or heating cycle. For this reason, we were not able to replace the AFM tip and complete the full hysteresis loop.

We overlay the average THz near-field signal with DC conductance measured in the same apparatus on the same film (green). The several orders of magnitude jump in DC conductance, signifying the insulator-metal transition, occurs to within 1 K of the same temperatures as the factor of 2 jump in the THz near-field signal. The slight offset in the THz near-field signal transition temperature compared to the transport transition temperature can be understood as due to several effects. First, we would expect local imaging experiments to record higher average near-field signal at temperatures below the DC transport transition because the formation of unconnected metallic domains precedes the percolative transition forming a continuous conducting path through the sample. Second, we would expect the THz near-field signal to increase at temperatures below the DC transport transition as our theory predicts: the slight increase in the insulating state THz conductivity leads to a rather large THz near-field signal.

Supplementary Note 3: Autocorrelation analysis of AFM resolution

The custom AFM tips used for THz-SNOM limit the AFM resolution in THz measurements. Supplementary Figure 3a shows a typical AFM image of the VO_2 film taken with the THz-SNOM.

The grains in the film appear larger than in the MIR-SNOM (Supplementary Figure 3b) due to the increased radius of the THz-SNOM tip. We quantify the difference in AFM resolution by performing an autocorrelation analysis of the two AFM images. The full width at half maximum (FWHM) of the autocorrelation curve determines the average feature size of an AFM image. The THz AFM has an autocorrelation FWHM of 134nm (Supplementary Figure 3d, red line), whereas the MIR AFM reveals an average VO_2 grain size of 50nm.

Supplementary Figure 3c show a MIR near-field image of metallic domains in an insulating background at 342K. These domains are composed of many individual grains. A similar analysis of this image obtains an autocorrelation peak FWHM of 106nm (Supplementary Figure 3d, black line), which we can assign to the average size of metallic domains. Thus, the THz-AFM has sufficient resolution to resolve the metallic domains at some point in the transition.

Supplementary Note 4: Width of the IMT in MIR near-field images

In Supplementary Figure 4 we plot the difference in the MIR signal levels between the insulating and metallic states at each temperature for which there is coexistence. The coexistence region spans from 342 K to 350 K and is a good indication of the width of the transition in temperature.

Supplementary Note 5: Low-frequency reflectivity of an incoherent metal

The Drude model provides a consistent basis for discussing the frequency dependence of material optical response. The dielectric function $\tilde{\epsilon} = \epsilon_1 + i\epsilon_2$ is given by the Drude model as

$$\epsilon_1 = 1 - \omega_P^2 \tau^2 \frac{1}{1 + \omega^2 \tau^2} \tag{3}$$

$$\epsilon_2 = \frac{\omega_P^2 \tau}{\omega} \frac{1}{1 + \omega^2 \tau^2} \tag{4}$$

where ω_P and τ are the plasma frequency and scattering time, respectively. For simplicity we consider the reflectivity of a material in vacuum at normal incidence, which is

$$R = \left| \frac{\tilde{n} - 1}{\tilde{n} + 1} \right| \tag{5}$$

where $\tilde{n} = n + ik$ is the complex index of refraction of the material. When the magnetic permiability of the material is equal to 1, the index of refraction is related to the optical permitivitty as follows:

$$n^2 = \frac{\sqrt{\epsilon_1^2 + \epsilon_2^2} + \epsilon_1}{2} \tag{6}$$

$$k^2 = \frac{\sqrt{\epsilon_1^2 + \epsilon_2^2} - \epsilon_1}{2}. \tag{7}$$

Consider the case of an incoherent metal where $\omega_P \tau \approx 1$ in the low frequency limit $\omega \tau \ll 1$. This represents a material with a low but finite conductivity, as for the high $t_\perp / U$ case in the insulating state (main text). In this limit we obtain the following approximations:

$$\epsilon_2 \approx \frac{\omega_P^2 \tau}{\omega} \tag{8}$$

$$n \approx k \approx \frac{\sqrt{\epsilon_2}}{2} \tag{9}$$

Substituting Eqs. 8 and 9 into Eq. 5, we obtain

$$R \approx \frac{\frac{\omega_P}{\omega} - \sqrt{2\frac{\omega_P}{\omega}} + 2}{\frac{\omega_P}{\omega} + \sqrt{2\frac{\omega_P}{\omega}} + 2} \tag{10}$$

This function is plotted in Supplementary Figure 5 for $\omega_P = 1/\tau = 5000\text{cm}^{-1}$ along with the full Drude expression for reflectivity. At low frequencies, the reflectivity begins to increase rapidly, approaching 1 for $\omega = 0$.

Supplementary Note 6: Generating near-field histograms from dimer Hubbard model calculations

Phase separation in granular VO_2 films is likely due to local variations in T_c caused by long-range interactions such as strain, local stoichiometric variations, or long-range Coulomb interactions[1,2]. This is equivalent to having a material where T_c is homogenous, but there are local variations in the temperature of the film. We use the calculated near-field signal as a function of temperature to model the near-field signal distribution that would be acquired in an imaging experiment. For each temperature T_j we generate an N-element vector V of temperatures that are normally distributed about T_j with a standard deviation of 1K. In Figs. 5a and 5c of the main text, we plot the histogram

of $S(V) + \delta S$, where $S(x)$ is the near-field signal calculated at temperature x, and δS is a random white noise term. The white noise is added at each pixel to simulate the experimental error in detected near-field signal due to electronic and environmental background.

Supplementary References

1. Jamei, R., Kivelson, S. & Spivak, B. Universal aspects of Coulomb-frustrated phase separation. *Phys. Rev. Lett.* **94**, 056805 (2005).

2. McLeod, A. S. *et al.* Nanotextured phase coexistence in the correlated insulator V_2O_3. *Nature Physics* **13**, 80 (2016).

3. Najera, O. & Rozenberg, M. Multiple crossovers and coherent states in a Mott-Peierls insulator. *Physical Review B* **97**, 045108 (2018).

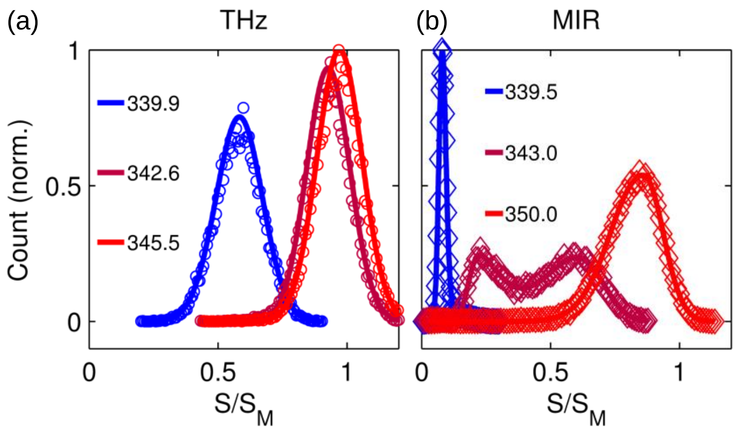

Supplementary Figure 1: Three representative histograms of near-field images taken at THz (a) and MIR (b) frequencies. The open symbols are the raw histogram bin heights, and the solid lines are best fits to the functions outlined in the text.

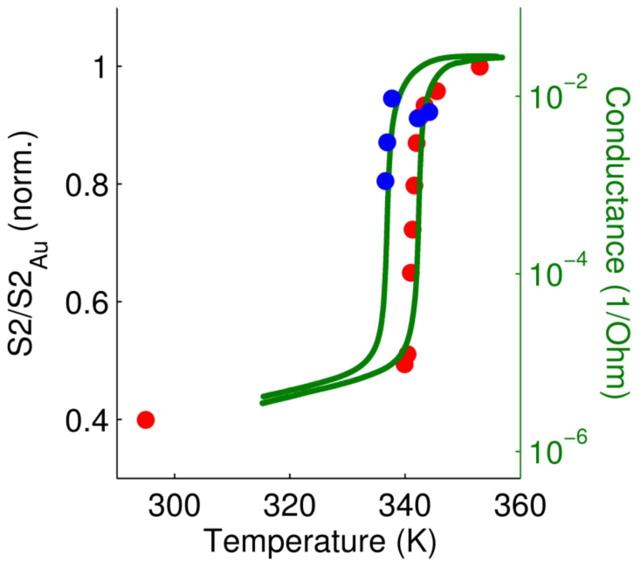

Supplementary Figure 2: THz near-field signal transition and DC transport transition measured on the same film in the same apparatus. Circles are the average THz near-field signal at each temperature normalized to the near-field signal on gold (left axis). Red circles correspond to temperatures during the warming cycle, while blue circles correspond to temperatures during the cooling cycle. The green line is the DC conductivity measured on the VO_2 film in the THz-SNOM apparatus as a function of temperature (right axis).

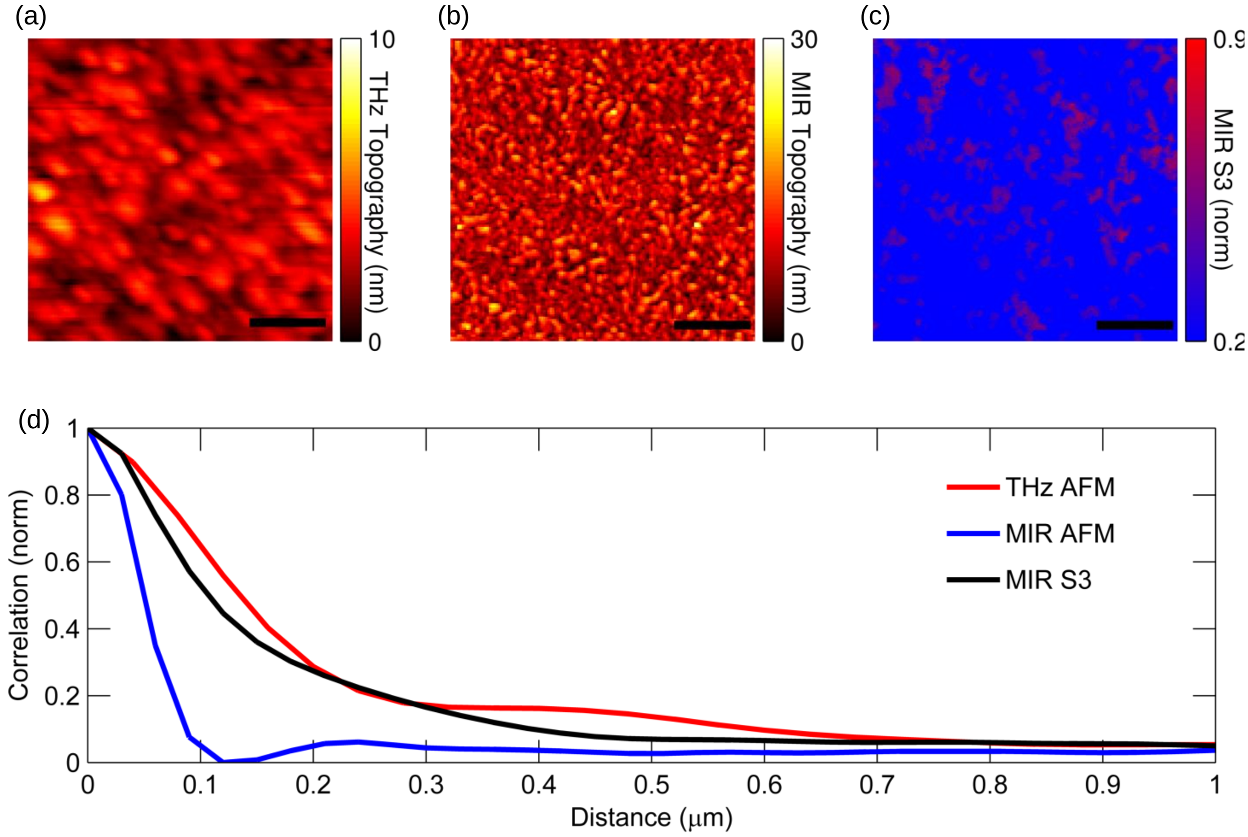

Supplementary Figure 3: Analysis of the THz-SNOM spatial resolution. (a) THz-SNOM AFM image at 342 K. (b) MIR-SNOM AFM image at 342K. (c) MIR-SNOM S3 image of VO_2 metallic domains at 342K. Scale bar in all images is 1 μm. (d) 2D autocorrelation curves for the three images. The width of the peak at the center of the autocorrelation corresponds to the size of features in the images.

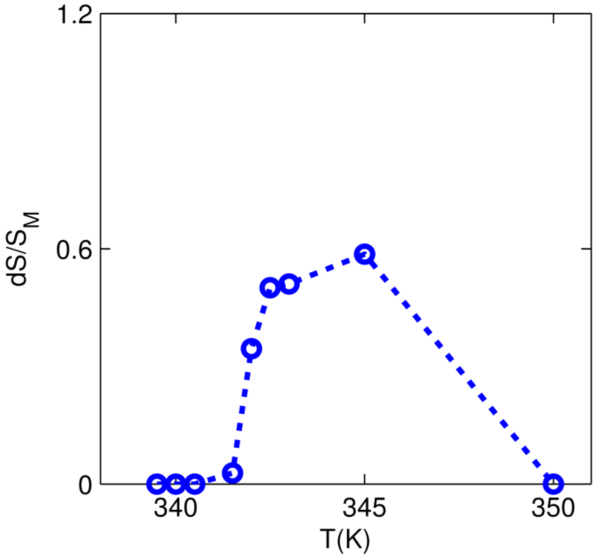

Supplementary Figure 4: Difference in average MIR near-field signal between the insulating and metallic states, as a function of temperature, normalized to the MIR near-field signal level in the metallic state.

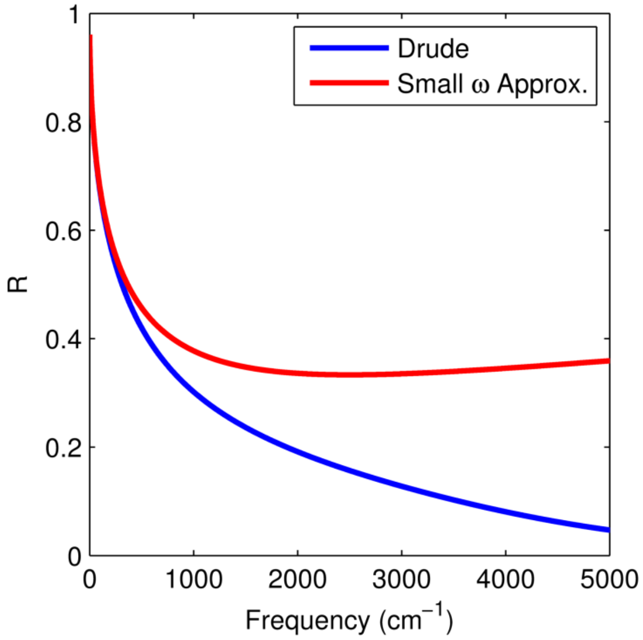

Supplementary Figure 5: Theoretical reflectivity of a Drude material with $\omega_P = 1/\tau = 5000\text{cm}^{-1}$ as calculated with the full Drude model or with Eq. 10.

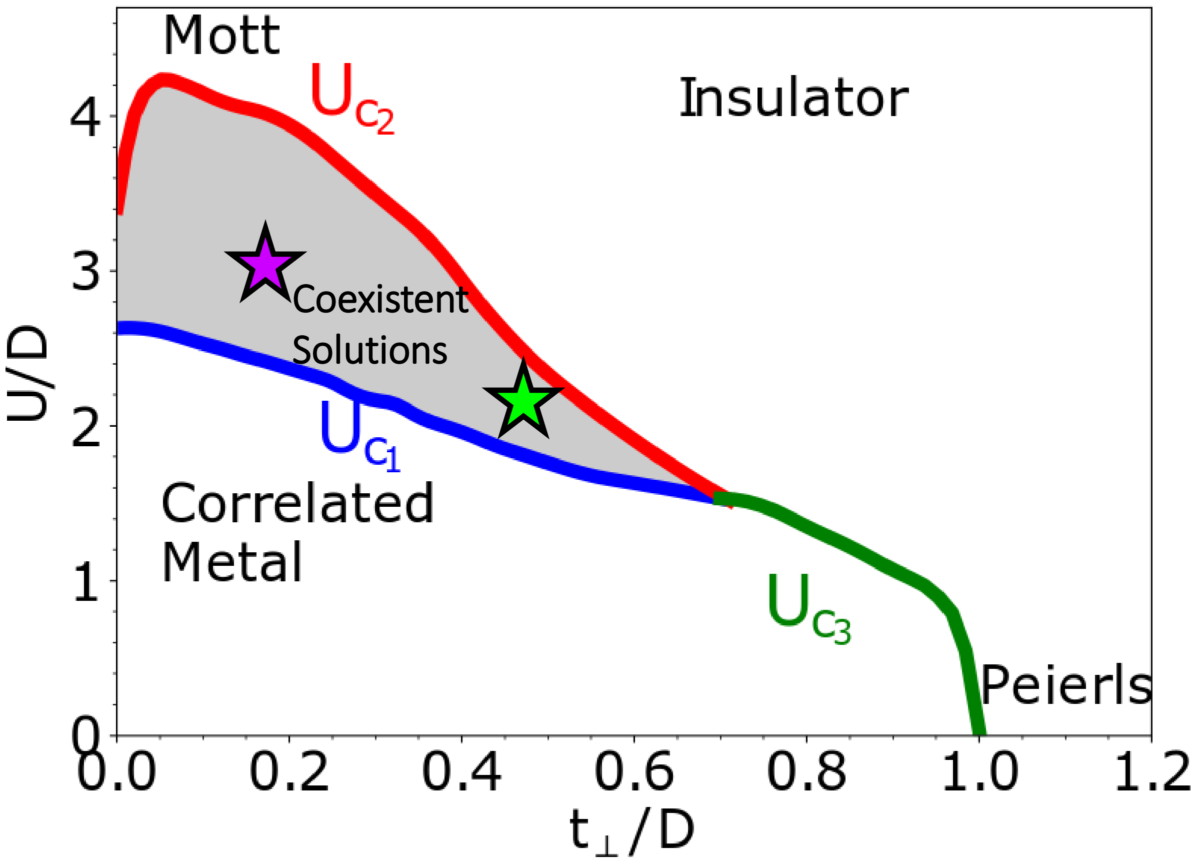

Supplementary Figure 6: Phase diagram of the dimer Hubbard model, reproduced from Ref. [3]. The purple star corresponds to the low $t_\perp/U$ case, and the green star corresponds to the high $t_\perp/U$ case, as discussed in the main text. Both cases are within the coexistence regime of the model.
